# Supplementary material for: Iterative improvement in the automatic modular design of robot swarms
Source: PeerJ Comput Sci. 2020 Dec 7;6:e322. doi: 10.7717/peerj-cs.322 (PMC7924708; doi:10.7717/peerj-cs.322)
Supplement: Supplemental Information 3 [file peerj-cs-06-322-s003.zip › argos3/doc/api/standalone/a00645_source.html]

ARGoS: plugins/simulator/visualizations/qt-opengl/qtopengl\_lua\_find\_dialog.h Source File


- Main Page
- Related Pages
- Namespaces
- Classes
- Files

- File List
- File Members

# plugins/simulator/visualizations/qt-opengl/qtopengl\_lua\_find\_dialog.h

Go to the documentation of this file.

```
00001 
00007 #ifndef QTOPENGL_LUA_FIND_DIALOG_H
00008 #define QTOPENGL_LUA_FIND_DIALOG_H
00009 
00010 namespace argos {
00011    class CQTOpenGLLuaFindDialog;
00012 }
00013 
00014 #include <QDialog>
00015 
00016 class QCheckBox;
00017 class QDialogButtonBox;
00018 class QGroupBox;
00019 class QLabel;
00020 class QLineEdit;
00021 class QPushButton;
00022 
00023 namespace argos {
00024 
00025    class CQTOpenGLLuaFindDialog : public QDialog {
00026 
00027       Q_OBJECT
00028 
00029    public:
00030 
00031       CQTOpenGLLuaFindDialog(QWidget *parent = 0);
00032 
00033    private:
00034 
00035       QLabel* m_pcLabel;
00036       QLineEdit* m_pcLineEdit;
00037       QCheckBox* m_pcCaseCheckBox;
00038       QCheckBox* m_pcFromStartCheckBox;
00039       QCheckBox* m_pcWholeWordsCheckBox;
00040       QCheckBox* m_pcSearchSelectionCheckBox;
00041       QCheckBox* m_pcBackwardCheckBox;
00042       QDialogButtonBox* m_pcButtonBox;
00043       QPushButton* m_pcFindButton;
00044       QPushButton* m_pcMoreButton;
00045       QWidget* m_pcExtension;
00046 
00047    };
00048 
00049 }
00050 
00051 #endif
```

---

Generated on 10 Jul 2018 for ARGoS by 
 1.6.1
